# Supplementary material for: Explainable detection of adverse drug reaction with imbalanced data distribution
Source: PLoS Comput Biol. 2022 Jun 15;18(6):e1010144. doi: 10.1371/journal.pcbi.1010144 (PMC9239481; doi:10.1371/journal.pcbi.1010144)
Supplement: Attachment — Submitted filename: Response to Reviews.pdf [file pcbi.1010144.s001.pdf]

Responses to the comments of reviewer 1

The authors proposed a new machine learning model for Adverse Drug Reaction detection, especially focused on the imbalance issue. They showed promising results based on this model. The reviewer appreciates the novelty of the work as well as the clarity of writing. But there are a few questions that need to be answered before it can be published:

1. **While the key component of the proposed model is CRF, the authors have not explained CRF in detail.**

Ans:

We have explained the CRF in more detail. (see *line 319, page 13*)

2. Could you explain why the proposed model consist of an LSTM followed by the BERT module? To the reviewer's understanding, BERT itself is able to handle the sequential information.

***Ans*:**

This is a very interested question. The ability of BERT to handle the sequential encoding mainly depends on position embeddings, which are added with token embeddings and segment embeddings as inputs to the BERT model. With the increase of the number of layers, the relevant sequence information will slightly dissipate in the output layer. For the conventional sequence labeling task, the use of LSTM followed by the BERT encoder can enhance the sequential information. Especially when the output classifier is CRF, such a combination can provide a better context information to support the implementation of CRF.

We conduct an ablation study to evaluate the effect of LSTM. The result show that the removal of LSTM will degrade the performance of the model. (see *line 183, page 8*)

3. Why the train/test splits are different for Tweets and Pubmed data?

***Ans*:**

To fairly compare with the previous studies, we conduct experiments on two corpora with different split strategies for the train/test sets. According to Cocos et al. [15], the **Twitter** datasets were split into a training and test set with a 3:1 ratio. For the **PubMed**, the previous study [32] suggests a ratio of 8:1:1 for the training/dev/test sets. The explanation was provided. (see *line 69, page 3*)

4. Fig. 2a and 2c are confusing to the reviewer. Shouldn't the performance converge to a specific value as the epochs increase? If the model is overfitted with large epochs, you should add regularization/dropout to make it converge, instead of using an epoch that is not converged.

***Ans*:**

For the training process of the CRF model, only the negative likelihood loss and accuracy score can be monitored. The negative likelihood can be minimized and converged to

a specific value as the epochs increase. In addition, the problem of unbalanced data invalidates the accuracy score, as discussed in the Introduction section. Thus, the viable solution is to specify training epochs for the proposed model, and then calculate F_1-score to select the best result.

The code of this paper is available at: https://github.com/wangjin0818/adverse_drug_reaction.

5. Equation 12 might be misleading, dash '-' is not distinguishable with minus sign.

***Ans*:**

The typo has been fixed.

Responses to the comments of reviewer 2

The objective of this paper is to tackle the data imbalance issue in sequence labeling tasks. Instead of using the conventional softmax in the output layer, a weighted variant of Conditional Random Field (CRF) is proposed to capture the relationship of labels between tokens. Experiments show that the CRF layers improve the existing sequence modeling architectures in two Adverse Drug Reactions (ADR) tasks.

Strengths:

1. **I think the data imbalance problem in machine learning in general is interesting. This work focuses specifically on the sequence labeling tasks in Natural Language Processing (NLP). This work might have a greater impact if extended to address the data imbalance problem in other data types such as time-series (e.g., speech).**

2. **The authors did a good job in summarizing imbalanced learning techniques in the related works and classified them into data-level methods and algorithm-level methods.**

3. **Table 3 in the experiments is extensive. That includes comparison of using softmax or CRF in the output layer of several existing NLP architectures. The experiments did show that the weighted version of CRF slightly improves across 3 metrics (precision, recall and F1-score) on both tasks (Twitter and PubMed).**

Weaknesses:

1. **I am not certain if this work fits into the theme of PLOS Computational Biology. Fundamentally, this work is purely NLP. My honest thought is other NLP-focused journals/conferences might be more suitable.**

Ans:

Developing an intelligence ADR detection system will assist drug design. This monitoring has traditionally been accomplished by surveying users. However, recent approaches have focused on automatic identification and extraction of ADR symptoms. The automatically recording ADR of users in social media can greatly help biopharmaceutical enterprises to improve their products. The explanation was provided. (*see line 7, page 2*)

2. **After a quick check in the Internet, I found that the idea of putting CRF on top of existing NLP architectures is not new. To name a few:**

   - (Huang et al., 2015) Bidirectional LSTM-CRF Models for Sequence Tagging, https://arxiv.org/abs/1508.01991

   - (Souza et al., 2019) Portuguese Named Entity Recognition using BERT-CRF, https://arxiv.org/abs/1909.10649

   - PyTorch tutorial on a combination of Bi-LSTM and CRF: https://pytorch.org/tutorials/beginner/nlp/advanced_tutorial.html

Ans:

Yes, but their tasks usually performed on the corpora with a balanced data distribution,

e.g., CoNLL, OntoNotes and WNUT. Conversely, we focus on performing ADR tasks on the corpora with an extremely imbalanced distribution. As described in Section 1, this may lead to a result that the CRF may classify all tokens to *O*. More evidence can be found in Section 4, showing that applying a weighted loss function performed better than the conventional CRF. The motivation of this paper is to propose a reweighting mechanism to improve the CRF. So that the minority classes, e.g., *ADR* or *Indication*, could be detected.

In particular, the CRF is a variant of Hidden Markov Model. The training process will depend on the Viterbi algorithm, which is a dynamic programming to ensure that the scores of all tokens are globally optimized. The introduction of a weighted loss function will completely change the solution process of the back propagation. Therefore, the main contribution of this paper is to conduct a viable solution for the weighted CRF model and apply it on the ADR task, instead of simply stacking the CRF on top of existing NLP architectures.

3. **There is no mentioning that if the implementation/code will be released for reproducibility. What is the choice of deep learning framework to implement the models (e.g., PyTorch or TensorFlow or something else)?**

Ans:

The code of this paper is available at: https://github.com/wangjin0818/adverse_drug_reaction.
